# Combinational risk factors of metabolic syndrome identified by fuzzy neural network analysis of health-check data

**DOI:** 10.1186/1472-6947-12-80

**Published:** 2012-08-01

**Authors:** Yasunori Ushida, Ryuji Kato, Kosuke Niwa, Daisuke Tanimura, Hideo Izawa, Kenji Yasui, Tomokazu Takase, Yasuko Yoshida, Mitsuo Kawase, Tsutomu Yoshida, Toyoaki Murohara, Hiroyuki Honda

**Affiliations:** 1 School of Engineering, Nagoya University, Furo-cho, Chikusa-ku, Nagoya, 464-8603, Japan; 2 NGK Insulators, Ltd, Sudacho, Mizuho-ku, Nagoya, 467-8530, Japan; 3 Nagoya University School of Medicine, Tsurumaicho, Showa-ku, Nagoya, 466-8550, Japan; 4 NGK Health Insurance Society, Sudacho, Mizuho-ku, Nagoya, 467-8530, Japan; 5 Aoyama Clinic, Sakae 3-7-13, Naka-ku, Nagoya, 460-0008, Japan; 6 MEXT Innovative Research Center for Preventative Medical Engineering, Furo-cho, Chikusa-ku, Nagoya, 464-8601, Japan; 7 Faculty of Pharmacy, Meijo University, Yagotoyama 150, Tenpaku-ku, Nagoya, 468-8503, Japan

**Keywords:** Data mining, Combinational risk factor, Fuzzy neural network, Glutamyltranspeptidase, Lifestyle disease, Personalized diagnostic method, White blood cell

## Abstract

**Background:**

Lifestyle-related diseases represented by metabolic syndrome develop as results of complex interaction. By using health check-up data from two large studies collected during a long-term follow-up, we searched for risk factors associated with the development of metabolic syndrome.

**Methods:**

In our original study, we selected 77 case subjects who developed metabolic syndrome during the follow-up and 152 healthy control subjects who were free of lifestyle-related risk components from among 1803 Japanese male employees. In a replication study, we selected 2196 case subjects and 2196 healthy control subjects from among 31343 other Japanese male employees. By means of a bioinformatics approach using a fuzzy neural network (FNN), we searched any significant combinations that are associated with MetS. To ensure that the risk combination selected by FNN analysis was statistically reliable, we performed logistic regression analysis including adjustment.

**Results:**

We selected a combination of an elevated level of γ-glutamyltranspeptidase (γ-GTP) and an elevated white blood cell (WBC) count as the most significant combination of risk factors for the development of metabolic syndrome. The FNN also identified the same tendency in a replication study. The clinical characteristics of γ-GTP level and WBC count were statistically significant even after adjustment, confirming that the results obtained from the fuzzy neural network are reasonable. Correlation ratio showed that an elevated level of γ-GTP is associated with habitual drinking of alcohol and a high WBC count is associated with habitual smoking.

**Conclusions:**

This result obtained by fuzzy neural network analysis of health check-up data from large long-term studies can be useful in providing a personalized novel diagnostic and therapeutic method involving the γ-GTP level and the WBC count.

## Background

Metabolic syndrome (MetS) is characterized by a clustering of metabolic abnormalities, including glucose intolerance, insulin resistance, central obesity, dyslipidemia, and hypertension, and it has been identified as a frequent cause to the development of cardiovascular disease [1]. The prevalence of MetS in Japan has increased over recent decades as a result of changes in diet and physical activity [2]. To investigate the relationship between diet or physical activity and risk marker plays effective roles in finding the most suitable lifestyle factor to improve developing MetS. It is useful for proposing a personalized diagnostic and therapeutic method. There is also an urgent need to establish an appropriate and sensitive screening marker to identify individuals at a high risk of developing MetS, thereby preventing a further increase in its incidence. So far, indices such as the low-density lipoprotein (LDL) to high-density lipoprotein (HDL) ratio (L/H) [3] or the ratio of adiponectin to homeostasis model assessment–insulin resistance (adiponectin/HOMA-IR ratio) [4] have been proposed as combinational risk factors. We have also reported that a combination of adiponectin receptor 1 (*ADIPOR1*; rs1539355) with an environmental factor (smoking habit) is suitable as a combinational risk factor for MetS [5]. There is, however, a need to identify new combinational risk factors.

In this study, we used a fuzzy neural network (FNN) in a bioinformatics approach to search for complex risk characteristics. Hirose et al. predicted the prevalence of MetS using artificial neural network [6]. The FNN is one of artificial neural network models that have been used in medical research as a powerful tool for the accurate detection of causal relationships [7-10]. FNN analysis has two main advantages. The first is its ability to select parameters on the basis of a parameter-increase method to permit the identification of the most influential parameters in the data. FNN analysis has the same predictable ability as multiple logistic regression. The second is its ability to extract predictive rules called fuzzy rule that can predict objective properties to reproduce the results.

So far, FNN has shown considerable flexibility in modeling of such complex phenomena as biochemical engineering processes (modeling of links between process valuables and process outputs) [11,12], food science (modeling of links between chemical components and sensory evaluation) [13], protein structural science (modeling of links between amino acid sequences and enzyme function) [14], housing science (modeling of links between physical environmental factors and human sensory evaluations) [15], and peptide science (modeling of links between peptide sequences and peptide functions) [16,17]. We therefore conjectured that FNN might serve as a suitable method for identifying specific characteristics that affect the pathogenesis of MetS.

The present study had two chief merits. The first was that the studies were based on subjects receiving health check-ups rather than on clinical patients; this has the advantage that periodical health examination is free of model bias, so that our results apply to the general population. The second merit was the high quality of our data because the relevant studies involved large numbers of subjects who were followed over a long time (at least seven years).

Overall, the aim of our studies was to identify reliable combinational risk factors associated with MetS by using an FNN and to contribute to the prevention of MetS by mitigating the identified risk combination.

## Methods

### Study design

To identify a significant combination of factors associated with MetS, we performed a two-stage study. The clinical characteristics before study start are summarized in Table1 and 2. In the original study, we selected 77 case subjects and 152 healthy control subjects from among 1803 Japanese male employees [5]. This longitudinal study was conducted by using health check-up data collected during a long-term follow-up (at least seven years). A replication study involved 2196 case subjects and 2196 healthy control subjects from among another 31343 other Japanese male employees. This study was also a longitudinal one and was conducted over eight years. All studies were performed according to the guidelines of the Declaration of Helsinki. Informed consent was obtained from all participants, and the studies were approved by Nagoya University School of Medicine. In both studies, we used clinical data before follow-up to predict the cause of MetS. 

**Table 1:** **Characteristics oforiginal study**

**Characteristic**	**n**	**Case mean ± SD or n (%)**	**n**	**Healthy control mean ± SD or n (%)**	***P *value**
Male n (%)	77	77 (100)	152	152 (100)	1.000
Age (years)	77	31.4 ± 7.5	152	30.6 ± 4.6	0.299
Height (cm)	77	172.1 ± 5.4	152	170.9 ± 5.5	0.125
Weight (kg)	77	68.2 ± 5.8	152	59.9 ± 6.3	8.28 × 10^−19^
BMI (kg/m^2^)	77	23.0 ± 1.4	152	20.5 ± 1.9	1.58 × 10^−21^
Systolic blood pressure (mmHg)	75	130.0 ± 12.5	152	116.9 ± 11.2	7.45 × 10^−14^
Diastolic blood pressure (mmHg)	75	78.4 ± 9.2	152	69.8 ± 7.0	1.92 × 10^−13^
Serum total cholesterol (mg/dl)	57	187.5 ± 28.5	99	166.0 ± 21.5	3.37 × 10^−7^
Serum triglycerides (mg/dl)	57	166.0 ± 148.7	98	74.1 ± 34.9	2.74 × 10^−8^
Serum HDL-cholesterol (mg/dl)	54	48.0 ± 11.0	90	57.1 ± 9.8	9.14 × 10^−7^
Fasting plasma glucose (mg/dl)	53	92.2 ± 9.7	85	88.2 ± 7.9	9.60 × 10^−3^
Alcohol habit n (%)	76	63 (82.9)	152	131 (86.2)	0.513
Smoking habit n (%)	75	51 (68.0)	151	81 (53.6)	3.94 × 10^−2^

Data are mean ± SD or n (%) unless noted otherwise.

Differences in characteristics between case and healthy control subjects were evaluated by linear regression analysis.

**Table 2:** **Characteristics ofreplication study**

**Characteristic**	**n**	**Case mean ± SD or n (%)**	**n**	**Healthy control mean ± SD or n (%)**	***P *value**
Male n (%)	2196	2196 (100)	2196	2196 (100)	1.000
Age (years)	2196	43.5 ± 7.7	2196	43.4 ± 5.4	0.519
Height (cm)	2196	170.3 ± 5.7	2196	169 ± 5.8	1.58 × 10^−13^
Weight (kg)	2196	72.4 ± 9.1	2196	60.4 ± 6.7	<1.0 × 10^−99^
BMI (kg/m^2^)	2196	24.9 ± 2.7	2196	21.1 ± 1.9	<1.0 × 10^−99^
Systolic blood pressure (mmHg)	2194	125 ± 13.7	2196	110.4 ± 9.4	<1.0 × 10^−99^
Diastolic blood pressure (mmHg)	2195	79.9 ± 10.0	2196	69.3 ± 7.6	<1.0 × 10^−99^
Serum total cholesterol (mg/dl)	2196	204.9 ± 34.8	2196	186.7 ± 30.4	3.10 × 10^−73^
Serum triglycerides (mg/dl)	2196	161.4 ± 115.6	2196	76.1 ± 28.0	<1.0 × 10^−99^
Serum HDL-cholesterol (mg/dl)	2196	55.0 ± 13.3	2196	67.6 ± 14.8	<1.0 × 10^−99^
Fasting plasma glucose (mg/dl)	2196	99.7 ± 19.9	2196	90.1 ± 7.4	1.28 × 10^−95^
Alcohol habit n (%)	2194	1693 (77.2)	2195	1704 (77.6)	0.712
Smoking habit n (%)	2195	1427 (65.0)	2195	1195 (54.4)	8.17 × 10^−13^

Data are mean ± SD or n (%) unless noted otherwise.

Differences in characteristics between case and healthy control subjects were evaluated by linear regression analysis.

### Definitions of case, healthy control and normal control

We used the criteria proposed by the Japan Society for the Study of Obesity (JASSO) [18] to identify subjects with MetS and supercontrol subjects.

1) Obesity: Waist circumference ≥85 cm in men or body-mass index (BMI) ≥25 kg/m^2^ if the waist circumference was not measured.

2) Raised blood pressure: systolic blood pressure ≥130 mmHg and/or diastolic blood pressure ≥85 mmHg.

3) Dyslipidemia: triglyceride ≥150 mg/dL and/or HDL cholesterol <40 mg/dL

4) Raised fasting glucose: fasting glucose ≥110 mg/dL

Subjects were classified as suffering from MetS if they were obese and they showed any two of the other three criteria. Subjects who were free of any of the risk components were classified as supercontrol. Then we defined case, healthy control and normal control according to the criteria below.

Case: Subjects who developed MetS during the follow-up.

Healthy control: Subjects who were remained as supercontrol during the follow-up.

Normal control: Subjects who weren’t MetS during follow-up.

Subjects with antihypertensive, lipid-lowering and anti-diabetic agents were excluded from analysis.

### Measurements

The baseline health examination performed before follow-up included physical measurements, serum biochemical measurements, urine measurements, medication use and a questionnaire. Physical measurements of height, weight and body mass index were measured in the fasting state. Blood samples were obtained from subjects in the fasted condition for serum biochemical measurements. After the subject had rested for 10 min in sitting position, 14 ml of blood were collected from the antecubital vein into tubes containing EDTA. After blood samples were sent to the clinical laboratory testing company, biochemical measurements were determined according to standard laboratory procedures. Biochemical measurements collected in this study include;

(1) Lipids: total cholesterol, triglyceride and HDL-cholesterol.

(2) Carbohydrate: glucose.

(3) Non-protein nitrogenous compounds: urea nitrogen, creatinine and uric acid.

(4) Serum enzymes: γ-glutamyltranspeptidase (γ-GTP), glutamic-oxyacetic transaminase (GOT), glutamic-pyvuvic transaminase (GPT).

(5) Hematology: red blood cells (RBC), hemoglobin, hematocrit and white blood cells (WBC).

Urine samples were also collected in the morning. After urine samples were sent to clinical laboratory testing company, urine uribilinogen, urine protein, urine sugar and urinary occult blood were measured. Medication use was assessed by the examining physicians. Drinking habit and smoking habit were collected by standard questionnaire. The questionnaire asked about the frequency of alcohol consumption on a weekly basis and smoking habit (never, past or current smoker). Drinking habit was defined as the subject who drank once a week and more. Smoking habit was defined as past or current smoker. In replication study, exercise habit was also divided into four categories by the time of exercise per week; exercising every day, exercising twice or more a week, exercising once a week and no-exercising. The aim of our studies was to identify risk factors from routine health check-up parameters generally measured. Therefore, the well-defined risk factors such as insulin weren’t measured in our study.

### FNN analysis

**Figure 1:**
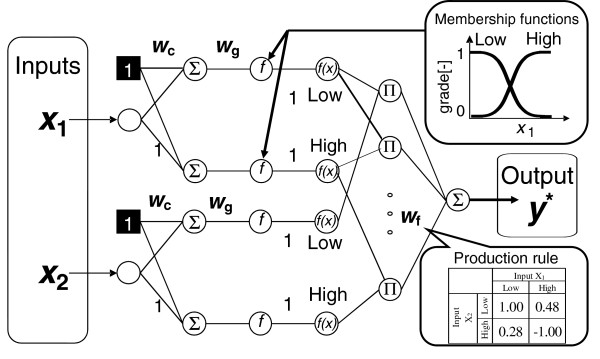
**Fuzzy neuralnetwork (FNN)model (twoinputs, oneoutput).** The most effective combination of input characteristic contributing to MetS was identified by the use of parameter-increasing method.

We conducted an FNN analysis to identify any significant combinations that are associated with MetS. The procedure for constructing the model is shown schematically in Figure1. This model has two inputs *x*_1_ and *x*_2,_ one output y*, and two membership functions *f*_low_ and *f*_high_ in each premise. FNN has three kinds of connection weights: *W*_c_*W*_g_, and *W*_f_[19]. The connection weights *W*_c_ and *W*_g_ determine the positions and gradients of the sigmoid functions; these decide the grade of each membership function *f*_low_ or *f*_high_ by means of the formula shown below,

(1) $$f\left(x\right)=1/\left[1+exp\left\{-W_{g}\left(x+W_{c}\right)\right\}\right]$$

where *x* is input value and *f(x)* is the product of the grade of membership function. The products of the grades are fed to the next unit Π. *W*_f_ is the weight of each production rule and decides the output y* by means of the sum of the connection weights *W*_f_ and Π. In our original study, for input data we used 16 clinical characteristics that were not directly related to MetS criteria (Table3). In our replication study, we used the two clinical characteristics that were identified as a result of the original study. All datasets were randomly arranged, divided equally into five datasets, and subjected to a fivefold cross-validation (CV) by using four datasets as training data and one dataset for validation. Through this fivefold CV, the combination of two input parameters that provided the best predictive accuracy as an average throughout the CV was selected by means of the forward-selection method. The accuracy was calculated as shown below, and the model with the highest accuracy was selected as the best combination.

(2) $$\text{Accuracy}\left(\%\right)=\left\{\left(\text{the number of correct estimation for training data}\right)/\left(\text{the number of training data}\right)\right\}\times 1/3+\left\{\left(\text{the number of correct estimation for test data}\right)/\left(\text{the number of test data}\right)\right\}\times 2/3$$

**Table 3:** **Sixteen inputcharacteristics forthe FNNanalysis**

**Input number**			
1	Smoking habit	9	Hematocrit (%)
2	Blood urea nitrogen (mg/dl)	10	RBC (million cells/μL)
3	Creatinine (mg/dl)	11	WBC (cells/μL)
4	Uric Acid (mg/dl)	12	Urine urobilinogen (%)
5	γ-GTP (IU/L)	13	Urine protein (%)
6	Hemoglobin (g/dl)	14	Urine sugar (%)
7	GOT (IU/L)	15	Urinary occult blood (%)
8	GPT (IU/L)	16	Alcohol habit

Here, we judged that a correct estimation was achieved if the output signal y* from the model was more than 0 for a case subject and less than 0 for a healthy control subject; otherwise, the estimation was judged to be incorrect. We compared the accuracy in FNN analysis with those in multiple linear regression and multiple logistic regression.

### Statistical analysis

To ensure that the risk combination selected by FNN analysis was statistically reliable, we performed logistic regression analysis involving a selected characteristic as an independent variable and the definition of “case subject” or “healthy control subject” as a dependent variable. Study estimates were adjusted for age, drinking habit, smoking habit and the components of MetS including BMI, systolic blood pressure, diastolic blood pressure, triglyceride, HDL-cholesterol and fasting plasma glucose. In replication study, we added exercise habit for adjustment. In addition, using correlation coefficient and correlation ratio, we tested the association between the selected characteristic and other clinical characteristics. A characteristic was considered statistically significant at a *P* value of less than 0.05. All statistical analyses were performed with R software (Version 2.13.1, http://www.r-project.org/).

## Results

### Clinical characteristics

The clinical characteristics before study start in the original study and in the replication study are listed in Table1 and 2, respectively. In both studies, the weight, BMI, systolic blood pressure, diastolic blood pressure, serum total cholesterol, serum triglyceride, and fasting plasma glucose were significantly higher in the case subjects than in the healthy control subjects, whereas serum HDL–cholesterol was significantly lower in the case subjects. In the replication study, the case subjects were significantly taller than the healthy control subjects.

### FNN analysis (original study)

**Figure 2:**
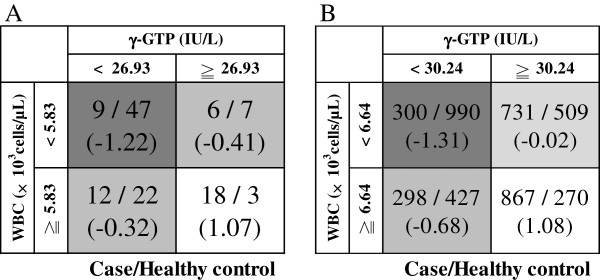
**Fuzzy rule.** The fuzzy rule visualizes the risk combination identified by FNN analysis. The numbers of case subjects and healthy control subjects are shown in the upper line of each cell of the matrix, and the weights required to yield a case of MetS are shown in the lower line of each cell of the matrix. In both studies, most case subjects were classified as showing high levels of γ-GTP and high WBC counts, giving a high weight of 1.07 or 1.08, which means a significant factor for a case of MetS. (**A**): Original study. (**B**): Replication study.

**Figure 3:**
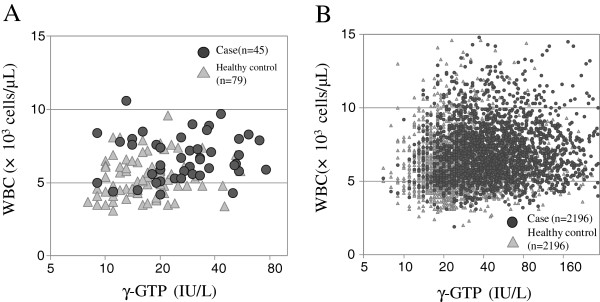
**Scatter plotsof γ-GTPlevel versusWBC count.** Scatter plots of γ-GTP level versus WBC count show that a combination of an elevated γ-GTP level and an elevated WBC count is associated with MetS. (**A**): Original study. (**B**): Replication study.

By means of the FNN analysis of health check-up data before study start from the original study (Table4), we identified a combination of the γ-glutamyltranspeptidase (γ-GTP) level and the white blood cell (WBC) count as being indicative of MetS. The FNN analysis had a high accuracy of 77.4% compared with the baseline of 63.7% calculated by the null method that estimates all subjects to be case subjects or healthy control subjects. This accuracy in FNN analysis was similar to an accuracy of 75.8% in multiple linear regression and an accuracy of 75.8% in multiple logistic regression. This combination of parameters, which showed the best predictive accuracy, is illustrated as a fuzzy rule in Figure2A. In this matrix, most case subjects were classified in the high γ-GTP level (≥26.9 IU/L) and high WBC count (≥5.83 × 10^3^ cells/μL) group, whereas most healthy control subjects were classified in the low γ-GTP level (<26.9 IU/L) and low WBC count (<5.83 × 10^3^ cells/μL) group. The numbers of case subjects and healthy control subjects are shown in the upper line of each cell of the matrix and the weights required to yield a case of MetS are shown in the lower line of each cell of the matrix. The matrix for the high γ-GTP level and high WBC count showed a high weight of 1.07, which corresponds to a significant factor for a case of MetS. This trend was also shown by a scatter plot of γ-GTP level versus WBC count (Figure3A). 

**Table 4:** **Inputs selectedby FNN**

**Study**	**Input number**	**Baseline (%)**	**FNN**	**Accuracy (%)**		**number of subject**		**Input characteristic**	
				**multiple linear regression**	**multiple logistic regression**	**Case**	**Healthy control**	**1 input**	**2 inputs**
Original Study	1 input	63.46	73.88	69.23	73.72	57	99	γ-GTP (IU/L)	−
	2 inputs	63.71	77.43	75.81	75.81	45	79	γ-GTP (IU/L)	WBC (cells/μL)
Replication Study	1 input	50.00	64.96	64.18	66.44	2196	2196	γ-GTP (IU/L)	−
	2 inputs	50.00	67.14	66.10	68.53	2196	2196	γ-GTP (IU/L)	WBC (cells/μL)

### FNN analysis (replication study)

The replication study confirmed the association between MetS development and a combination of a high γ-GTP level and a high WBC count. The FNN analysis showed a high accuracy of 67.1% compared with a baseline of 50.0%. This accuracy was similar to an accuracy of 66.1% in multiple linear regression and an accuracy of 68.5% in multiple logistic regression (Table4). In the fuzzy rule, most case subjects were classified as showing a combination of a high γ-GTP level (≥30.2 IU/L) and a high WBC count (≥6.64 × 10^3^ cells/μL), which corresponded to a high weighting of 1.08 (Figure2B). This fuzzy rule was also visualized as a scatter plot (Figure3B).

### Statistical verification of the γ-GTP level and WBC count as an indicator of MetS

Table5 shows the differences in the γ-GTP level and the WBC count between the case subjects and the healthy control subjects. The difference between γ-GTP level and MetS was significant after adjusting for age, drinking habit, smoking habit and the components of MetS (original study: *P* = 0.014, replication study: *P* = 1.71 × 10^−5^, combined study: *P* = 3.11 × 10^−6^). This significant difference remained significant after adjusting for exercising habit in replication study (*P* = 1.69 × 10^−5^). Although the difference between WBC and MetS was weak after adjusting for age, drinking habit, smoking habit and the components of MetS in replication study, the association remained significant in combined study (original study: *P* = 0.002, replication study: *P* = 0.107, combined study: *P* = 0.031). These results showed that the γ-GTP level and the WBC count, as selected by FNN analysis, together form a statistically reliable indicator.

**Table 5:** **Statistical analysisof characteristicsselected byFNN**

**Study**	**Characteristic**	**model 1**		**model 2**^**b**^		**model 3**^**c**^		**model 4**^**d**^	
		**OR (95%CI)**	***P *value**	**OR (95%CI)**	***P *value**	**OR (95%CI)**	***P *value**	**OR (95%CI)**	***P *value**
Original Study	γ-GTP (doubling)^a^	4.71 (2.63–8.41)	1.69 × 10^−7^	5.98 (3.12–11.5)	7.25 × 10^−8^	4.06 (1.33–12.4)	0.014		
	WBC (1000 cells/μL)	1.83 (1.39–2.41)	1.62 × 10^−5^	1.94 (1.41–2.65)	3.86 × 10^−5^	2.69 (1.44–5.02)	0.002		
Replication Study	γ-GTP (doubling)^a^	2.64 (2.45–2.85)	<1.0 × 10^−99^	2.84 (2.62–3.08)	<1.0 × 10^−99^	1.32 (1.17–1.51)	1.71 × 10^−5^	1.33 (1.17–1.51)	1.69 × 10^−5^
	WBC (1000 cells/μL)	1.31 (1.26–1.35)	2.73 × 10^−51^	1.30 (1.25–1.35)	5.06 × 10^−42^	1.05 (0.99–1.12)	0.107	1.06 (0.99–1.12)	0.094
Combined Study^e^	γ-GTP (doubling)^a^	2.65 (2.46–2.86)	<1.0 × 10^−99^	2.86 (2.64–3.10)	<1.0 × 10^−99^	1.35 (1.19–1.53)	3.11 × 10^−6^		
	WBC (1000 cells/μL)	1.32 (1.27–1.36)	1.28 × 10^−55^	1.31 (1.26–1.36)	1.62 × 10^−45^	1.07 (1.01–1.14)	0.031		

Odds ratio (OR) with 95 % confidence interval (CI) indicates the proportional change in risk associated with each increase by the amount indicated in parentheses.

^a^geometric mean (2.5–97.5 %) was used because of skewed distriburions.

Differences in characteristics between case and healthy control subjects were evaluated by logistic regression analysis.

^b^Differences were evaluated by logistic regression analysis with adjustment for age, drinking habit and smoking habit.

^c^Differences were evaluated by logistic regression analysis with adjustment for age, drinking habit, smoking habit and the components of MetS.

^d^Differences were evaluated by logistic regression analysis with adjustment for age, drinking habit, smoking habit, the components of MetS and exercise habit.

^e^In combined study, the difference of study was added in adjustment.

**Figure 4:**
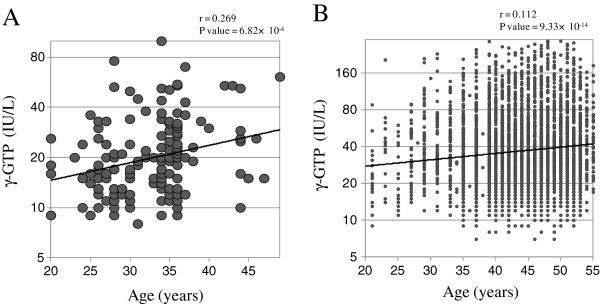
**Scatter plotsof ageversus γ-GTPlevel.** Scatter plots of age versus γ-GTP level show that the difference in the γ-GTP level between the original study and the replication study was due to the age of the subjects. (**A**): Original study. (**B**): Replication study.

To explain the difference between the γ-GTP levels in the original study (mean in healthy control subjects = 16.3 IU/L) and those in the replication study (mean in healthy control subjects = 27.3 IU/L), we compared the clinical characteristics of the participants in the two studies. We found that age had a significant effect (*P* < 1.0 × 10^–99^), so we investigated the correlation coefficient between age and γ-GTP levels. A scatter plot of age versus γ-GTP levels showed that the difference in the mean γ-GTP level was due to age (Figure4).

Finally, we calculated correlation ratio for the original study (Table6). The γ-GTP level was significantly associated with habitual drinking of alcohol (*P* = 1.41 × 10^−2^), but not with habitual smoking (*P* = 0.406). On the other hand, the WBC count was significantly associated with habitual smoking (*P* = 1.18 × 10^−5^), but not with habitual drinking (*P* = 0.695). The same tendency was found in the replication study (Table7). 

**Table 6:** **Correlation ratiosin originalstudy**

**Characteristic 1**	**Characteristic 2**	**n**	**η**	***P *value**
γ-GTP (IU/L)	Alcohol habit	155	0.197	1.41 × 10^−2^
γ-GTP (IU/L)	Smoking habit	153	0.068	0.406
WBC (cells/μL)	Alcohol habit	137	−0.034	0.695
WBC (cells/μL)	Smoking habit	135	0.369	1.08 × 10^−5^

**Table 7:** **Correlation ratiosin replicationstudy**

**Characteristic 1**	**Characteristic 2**	**n**	**η**	***P *value**
γ-GTP (IU/L)	Alcohol habit	4389	0.232	<1.0 × 10^−99^
γ-GTP (IU/L)	Smoking habit	4390	0.068	7.01 × 10^−6^
WBC (cells/μL)	Alcohol habit	4389	−0.074	9.74 × 10^−7^
WBC (cells/μL)	Smoking habit	4390	0.386	<1.0 × 10^−99^

## Discussion

In our study, we used an FNN as a computational method to analyze complex characteristics. The FNN analysis is a powerful machine-learning method for detecting, with maximal accuracy, significant combinations of characteristics that are associated with a particular attribute. By using an FNN, we identified that a combination of the γ-GTP level and the WBC count is a characteristic that is associated with MetS. As shown in Table4, the accuracy in FNN analysis was similar to the accuracy in multiple linear regression and the accuracy in multiple logistic regression. However, FNN analysis also has an ability to visualize the risk of the high γ-GTP level and high WBC count group easily using fuzzy rule as Figure2. The FNN also lacked statistical significance, so we reexamined selected characteristics by means of statistical analysis with suitable adjustments. The statistical results confirmed that the γ-GTP level and the WBC count are significant factors in MetS, confirming that the FNN has good predictive powers and is suitable for use in practical applications.

We excluded the characteristics included in judgments of metabolic syndrome. We firstly conducted FNN analysis including the components of metabolic syndrome, selecting a combination of triglycerides and WBC. However, we thought these components directly affect the prevalence of MetS. We aimed to search latent risk factors using remaining 16 clinical and laboratory characteristics. We showed that an elevated γ-GTP level and an elevated WBC count are combinational risk factors for MetS. γ-GTP is a marker of fatty liver disease and γ-GTP levels have been found to be associated with the prevalence of MetS in previous East Asian studies [20-22]. An increase in levels of liver enzymes may be related to excess deposition of fat in the liver. The WBC count is a marker of systemic inflammation and it has also been found to be associated with the prevalence of MetS in a previous study [23]. The WBC count is controlled by cytokines, especially interleukin-6 and interleukin-8 [24], and WBCs play a major role in inflammatory processes and in defending the body against infectious disorders. In addition, a previous study has shown that the mean WBC count increases with an increase in serum γ-GTP [25]; this implied that elevated γ-GTP levels might reflect subclinical inflammation. This result from our FNN method may show that this combination has a synergistic effect.

Our study also showed that habitual drinking is related to an elevated level of γ-GTP, in agreement with a previous study [26]. However, we also showed that there is no significant association between habitual drinking and MetS. Similarly, we showed that habitual smoking is linked to an increase in the WBC count. However, the association between habitual smoking and MetS was low significant. This tendency was the same that found in a previous study [27]. Although γ-GTP levels and WBC counts are generally included in blood tests performed during periodic health examinations, these characteristics have seldom been considered in risk assessment. Our result could be useful in a personalized risk-prevention method, advising people with elevated γ-GTP level and an elevated WBC count to improve their diet and physical activity.

In this study, completely healthy people were used as controls. We also conducted logistic regression analysis between case subjects who developed MetS during the follow-up and normal control subjects who weren’t MetS during the follow-up. In original study, including 77 case subjects and 597 normal control subjects, the significant association between γ-GTP and MetS wasn’t observed (*P* value = 0.213). On the other hand, the significant association between WBC and MetS was observed (*P* value = 4.76 × 10^−4^). In replication study, including 2196 case subjects and 27246 normal control subjects, the significant association between γ-GTP and MetS was observed (*P* value = 5.00 × 10^−21^). The significant association between WBC and MetS was also observed (*P* value = 1.41 × 10^−26^). Although our study couldn’t find significant association between γ-GTP and MetS in original study partly because of small sample, we showed significant associations in replication study consisted of large subjects. This result suggests that both of the association between γ-GTP and MetS and the association between WBC and MetS may be derived from the difference between those who will develop the overt clinical picture of metabolic syndrome and those who will develop some of its components without fulfilling the criteria for its diagnosis.

Our present study has several limitations however. First, for clinical data before the follow-up, we used a modified definition of MetS involving the BMI instead of the waist circumference; however, several studies have shown that the BMI is an equally effective characteristic as the waist circumference and it has been adopted for analyses of the association between the adiponection gene and metabolic traits, including MetS [28]. Secondly, we analyzed data from male subjects exclusively. This was because only one woman showed an indication of MetS among 2061 Japanese company employees in our original study. Although the potential bias was minimized by adjusting for age, drinking habit, and smoking habit, our findings may have limited value in the case of women. Thirdly, we could not subdivide drinking habit and smoking habit quantitatively. As in a previous study [26], the strength of risk of MetS may be related to the drinking status or the smoking status.

## Conclusions

We have shown that the combination of the γ-GTP level and the WBC count is the most significant risk factor associated with MetS. By using a statistical analysis adjusted by age, drinking habit, smoking habit and the components of MetS, we confirmed that the FNN analysis method is suitable for identifying combinations of factors associated with the risk of lifestyle diseases. Our results may be useful in providing a novel personalized diagnostic and therapeutic method, depending on the individual subject’s γ-GTP level and WBC count.

## Abbreviations

*ADIPOR1*: Adiponectin receptor 1; BMI: Body-mass index; CV: Cross-validation; FNN: Fuzzy neural network; γ-GTP: γ-Glutamyltranspeptidase; GOT: Glutamic-oxyacetic transaminase; GPT: Glutamic-pyvuvic transaminase; HOMA-IR: Homeostasis model assessment–insulin resistance; LDL: Low-density lipoprotein; :HDL: High-density lipoprotein; MetS: Metabolic syndrome; RBC: Red blood cells; WBC: White blood cells.

## Competing interests

The authors declare that they have no competing interests.

## Authors’ contributions

YU designed the statistical model, performed the data analyses, and wrote the manuscript. RK and HH supervised the analyses. DT, HI, YY, and HH conceptualized the study. KY and TY provided the study data. All the authors approved the final manuscript.

## Pre-publication history

The pre-publication history for this paper can be accessed here:

http://www.biomedcentral.com/1472-6947/12/80/prepub
